# When Does an Individual Accept Misinformation? An Extended Investigation Through Cognitive Modeling

**DOI:** 10.1007/s42113-022-00136-3

**Published:** 2022-05-11

**Authors:** David Borukhson, Philipp Lorenz-Spreen, Marco Ragni

**Affiliations:** 1 Department for Computer Science, University of Freiburg, Freiburg, Germany; 2 Center for Adaptive Rationality, Max Planck Institute for Human Development, Berlin, Germany; 3 Predictive Analytics, Technical University Chemnitz, Chemnitz, Germany

**Keywords:** Predictive modeling, Fake news detection, Socio-psychological theories, Hybrid cognitive models

## Abstract

A new phenomenon is the spread and acceptance of misinformation and disinformation on an individual user level, facilitated by social media such as Twitter. So far, state-of-the-art socio-psychological theories and cognitive models focus on explaining how the accuracy of fake news is judged on average, with little consideration of the individual. In this paper, a breadth of core models are comparatively assessed on their predictive accuracy for the individual decision maker, i.e., how well can models predict an individual’s decision before the decision is made. To conduct this analysis, it requires the raw responses of each individual and the implementation and adaption of theories to predict the individual’s response. Building on methods formerly applied on smaller and more limited datasets, we used three previously collected large datasets with a total of 3794 participants and searched for, analyzed and refined existing classical and heuristic modeling approaches. The results suggest that classical reasoning, sentiment analysis models and heuristic approaches can best predict the “Accept” or “Reject” response of a person, headed by a model put together from research by Jay Van Bavel, while other models such as an implementation of “motivated reasoning” performed worse. Further, hybrid models that combine pairs of individual models achieve a significant increase in performance, pointing to an adaptive toolbox.

## Introduction

Misinformation and disinformation, such as “fake news”, are phenomena reaching far back in the history of the media. It typically refers to intentionally or unintentionally false information presented in a way that is deliberately or accidentally designed to mislead people. In the relatively young information ecosystem of the internet, such pieces of information can achieve particularly high spread, especially through self-organized sharing on social media (Vosoughi et al., 2018). As a result, individual decisions to believe and share information have reached new importance, as they can be a micro-level driver of the scaled spread of false information and collectively even put democratic decision-making at risk (Lewandowsky et al., 2020). Especially in recent years, misinformation has returned to prominence in connection with political events such as the 2016 UK European Union membership referendum, the 2016 US presidential election (Allcott and Gentzkow, 2017), or during the ongoing COVID-19 pandemic (Cinelli et al., 2020). By now, numerous theories have emerged describing the spread and acceptance of misinformation in the media (e.g. Talwar et al., 2019; Zhou et al., 2020). Thus it appears a timely and relevant approach to systematically compare some prominent theories of acceptance of news as well as more general human reasoning models on prediction accuracy. There are also other reasons for a person believing or sharing a particular bit of news, such as its partisanship (Osmundsen et al., 2021) or familiarity with the reported content (Pennycook and Rand, 2020). If we better understand the motives and mechanisms of susceptibility to believe misinformation on an individual cognitive level, action can be taken to reduce acceptance and spread of false news (Lazer et al., 2018; Lorenz-Spreen et al., 2020). In the work at hand, rather than on their drives and intentions to share news reports, we focus on the accuracy of persons’ detection of misinformation.

Modeling cognitive processes has long been of interest for understanding human reasoning and many theories from different fields of psychology have been formalized into computational models (Fum et al., 2007). The methods used in this paper follow the premise that models need to *predict* a future output of an individual and not just reproduce data. We use a setting to evaluate the ability of a model to explain a particular individual’s behavior. For this, experimental data is used with an identical training and test set per participant. As our implemented models all only have a very limited number of parameters and no ability to store responses, this yields a rigorous measure of how well a participant’s decisions can be explained within a particular model. Comparing cognitive models by formalizing psychological theories and evaluation of their predictive performances has been done with different techniques: E.g., Bayesian hierarchical approaches (Scheibehenne et al., 2013) or choosing strategies among rule- and example-based processes (Herzog and von Helversen, 2018). The current work is based on a framework which is designed algorithmically model cognitive processes and resulting decisions by individual reasoners (Cognitive Computation for Behavioral Reasoning Analysis, CCOBRA (Ragni et al., 2019)). We thus reformulated the selected theories and present them in a format that is compatible with the CCOBRA approach.

Within research on cognitive reasoning, susceptibility to misinformation in news reports has been studied with different paradigms describing its spread (Del Vicario et al., 2016) and acceptance (Rampersad and Althiyabi, 2020). Some studies refer to rather simple implementations of cognitive reasoning models based on the correlation of measured features (Pennycook and Rand, 2019). However as experimental data on news reasoning tasks has only recently become available, there appears to be a dearth of empirical quantification for comparing decision-making models in this domain. To fill this gap, study news acceptance decision-making by systematically comparing the predictive power of different influential theories on an experimental dataset covering such decisions. The approach in the present paper provides more fine grained analysis, considerably extends, and combines results first presented in our paper (Borukhson et al., 2021).

The experimental datasets that we use to test and compare the predictive performance of all implemented theories were collected and published by Pennycook and Rand (2019). They contain information on accuracy judgments of a number of “fake news” and real news items, as well as about the individual test participants. Participant-specific data from these sets can be used to test news item reasoning hypotheses put forth by Pennycook and Rand (2019) about motivated reasoning, and to compare them to other, more general heuristic theories in the tradition of the Adaptive Toolbox of Gigerenzer and Selten (2002).

### Quantifying News Reports

How can one formally quantify information related to news reports and to allow predicting the effect it has on a human reasoner who is exposed to it?

The experimental data that this work is based upon, contains experiments with “news items”. This is a kind of news reports that typically appears on various much-used websites: A news item consists of an image and a header of one or two lines of text that convey a claim or message. This format makes their layout standardized to an extent sufficient to use them as stimuli in behavioral studies. Properties of the news items from the data are known from other experimental pretests that asked participants to evaluate political partisanship, other characteristics such as perceived familiarity and perceived importance of the items, thus quantitatively measuring some of their features. The content of the pictures however was not analyzed, as the primary focus is on news headline processing and introducing image recognition techniques appears to be beyond the scope. On the participant level, apart from demographic information, a score for cognitive reflection was measured. These measured features on the item and participant levels allow us to compare various computational realizations of competing theories for misinformation acceptance.

### Reasoning About Cognitive Models on News Item Acceptance

Do some reasoning models predict news item acceptance decisions by individuals better than others? Among different classes of reasoning theories (Cognitive Models, Reasoning by Heuristics), a selection of relevant models is presented below. For each model, first a theoretical description is provided. Then a mathematical formalization is shown that we call the *model’s expected prediction*. This is the function that, given some features of news item and of an experiment participant as parameters, was used for the respective model’s performance evaluation. Most of these models were originally published in more general, psychological terms. For each one, we chose an algorithmic resp. arithmetic formulation in consideration of the variables were measured in the respective experiment. The formulation thus is designed to represent each theory in the closest possible way given the experimental features.

Note that while for many models there exist numerous other variants in implementation, often more complicated and with higher capacity than the ones presented, the goal of this paper is not to calculate highest-performance specifications for the given models, but to study and compare general approaches to modeling the processing of misinformation.

## Experiments

The experiments used for evaluating the models were conducted by Pennycook and Rand (2019) and Pennycook et al. (2021). They comprise accuracy judgments of participants about individual news items, consisting of an image and a headline, a shape in which they typically appear in a social media environment. The methodical details for experimentation are further elaborated in the original authors’ publication (Pennycook et al., 2020).

In the context of modeling, we also refer to a news item presented to a participant as a **task**. When shown to the participant in an experimental setting as a visual stimulus, the presentation of the news item is accompanied by the question: “To the best of your knowledge, how accurate is the claim in the above headline?” to be answered with one of four options (“not at all accurate”, “not very accurate”, “somewhat accurate”, “very accurate”). The body of experimental data used for modeling then contains the features of each task, as well as the known features of every participant and the response given to each task by every participant.

### Experiment 1

**Fig. 1:**
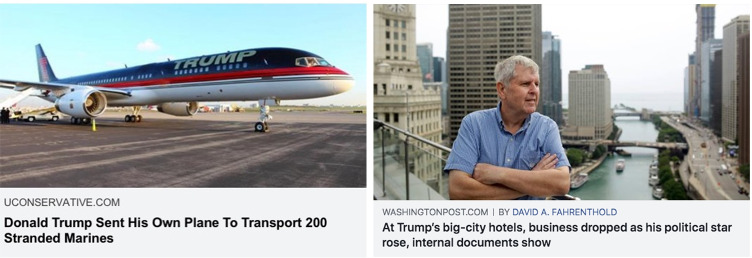
Two example news items from the dataset, as presented to participants. The layout of the other items is similar, only headlines and pictures differ

This experiment was completed as part of a study that took place in 2017 (Pennycook and Rand, 2019). 843 participants (763 with completed data) were recruited from Amazon Mechanical Turk. They were presented 20 partisan (10 Pro-Republican, 10 Pro-Democrat), and 10 neutral news items of which 15 were real news and 15 were fake news items and asked to rate them for accuracy. News items were compiled from a fact-checking website (for false information items) and mainstream news sources (for true items). Each item consists of a short headline underneath a picture; for an example see Fig. 1. They were presented to participants sequentially, in a random ordering. Apart from questions about demographics such as education, gender and age, participants were asked to complete a cognitive reflection test (CRT) consisting of seven questions. The CRT was devised to measure the tendency of a person to “resist reporting the first response that comes to mind” when presented with a question. The questions in a CRT typically hint at one solution that springs to mind at the first glance, but proves incorrect on second thought, e.g.: “A bat and a ball cost $1.10 in total. The bat costs $1.00 more than the ball. How much does the ball cost?” While $0.10 at first instance seems to be suitable, of course the correct answer is $0.05 (Frederick, 2005). In the experiments evaluated for this paper, a variant of the CRT with seven questions for logical reasoning was used.

The primary goal of this experiment was to compare the Motivated and Classical Reasoning accounts of processing news items. For Motivated Reasoning, CRT was expected to correlate positively with acceptance of fake news items of a partisanship corresponding to that of a test participant. However, in the analysis of the experiment, such correlation was not found: Instead CRT and accurate classification of news items were found positively correlated, thus hinting a Classical Reasoning explanation.

Data was included for the 763 participants who completed all stages of the experiment.^1^

#### Pretest

For this study, data from a pretest of 195 different persons was used, who were asked to judge on a scale to what extent an item was perceived as partisan for Republicans or Democrats (“more favorable to Democrats” vs. “more favorable to Republicans”) and whether the item appeared familiar or unfamiliar to the participant.^2^

**Relevant Measured Features** For each task, the following features relevant to models were measured:

- Perceived accuracy of headline (“To the best of your knowledge, how accurate is the claim in the above headline”, 1 to 4 scale),
- perceived familiarity of headline (“Have you ever seen or heard about this story before?”, 1 to 3 scale),
- reaction time for fake/real categorization response,
- CRT value as the mean of correct responses in all 7 CRT test questions (Thomson and Oppenheimer, 2016),
- conservatism of participant (1 to 7 scale) from the pretest in two separate questions on political ideology on social and economic issues,
- perceived political partisanship of news items (1 to 5 scale: “more favorable to Democrats” to “more favorable to Republicans”) from the pretest both as an absolute value and by partisanship of individuals,
- highest completed education level of the participant.

For perceived familiarity and partisanship of each news item, two values were provided respectively: One aggregated over all participants that were identified as rather Republican and one for Democrats. In all calculations below, the value corresponding to the partisanship of a given participant the model predicts for was used; individual partisanship was determined through a split over the mean of the dataset’s conservative scale. Where indicated, the mean value of Republican and Democrat aggregates was also taken into account.

### Experiment 2

This experiment was identical to Experiment 1, only the number of participants and the number and selection of news headlines used was different: 12 fake and 12 real news items were presented to each of the 2644 participants; complete data is available for 2546 participants. The study also included other questions such as about trust in media and fact-checkers, yet these are not relevant to the current models and research question. This Experiment took place in 2017 (Pennycook and Rand, 2019)^3^. Pretest and Relevant Measured Features are the same as in Experiment 1.

### Experiment 3

This study was conducted in 2019 through programmed online questionnaire forms (Pennycook et al., 2021). A set of fake news and real news items was compiled; these were to different extent favorable for supporters of the Republican party or for Democrats.

Each participant was presented 18 fake and 18 real headlines in a random order and asked to indicate whether they regarded them as true or false. The fake news headlines were selected from a fact-checking website that verified them as false; the real news came from mainstream media coverage that was contemporary to the selected misinformation items.

Subsequently participants answered a 7-item CRT test, followed by a number of demographic questions (such as age, gender and education). The study also included other conditions that involved different questions and primes (i.e., about news sharing behavior) instead of the question about perceived news item accuracy; these settings however are not relevant for the current research questions and were thus not further examined. Additionally, a Positive and Negative Affect Schedule (PANAS) score was taken: PANAS is a score that allows to measure self-reported positive and negative affectations independently (Watson et al., 1988). Conceptually it stems from the “Tripartite Model”, a model for anxiety and depression. It has been pointed out that more precisely the scale describes “activation of positively and negatively valenced affects” (Crawford and Henry, 2004; Watson et al., 1999). Here, PANAS appeared in the form of 20 questions asking to what extent the participant felt afraid, nervous, inspired, alert, et cetera.

Data for all 485 participants who completed every question of the study relevant to the present models was included in the evaluation.^4^

#### Pretest

For this study, data from a pretest from June 2019 was used where 3996 participants were asked to judge on a scale whether an item was more favorable for Republicans or Democrats. The same question was posed for (perceived) “familiarity”, “importance”, “worryingness” and “excitingness” of news items.^5^

**Relevant Measured Features** For each pair of participant and news item, the following were measured:

- Perceived accuracy of headline (“To the best of your knowledge, is this claim in the above headline accurate?”, Yes or No response),
- Reaction time for accuracy response,
- CRT value as the mean of correct responses in 6 CRT test questions.
- PANAS scores of the participant,
- conservatism of participant (1 to 5 scale),
- highest completed education level of participant.

Again, two aggregated values were provided respectively for (perceived) partisanship, “familiarity”, “importance”, “worryingness” and “excitingness” measures of each news item: One specified by the answers of Republican participants and one for Democrats. Similarly as for Datasets 1 and 2, the value corresponding to the partisanship of a currently modeled participant was used, the split of individual partisanship again imposed by the mean of the dataset’s conservative scale. Where indicated, these aggregates’ mean was too considered.

## Modeling

Given an individual and a model that to be trained, a A model’s **prediction** is “Accept” or “Reject”. Models attempt to maximize the number of predictions that correspond to the measured response of the same individual for each task.

The **expected prediction** is a probability value between 0 and 1 that represents the model’s chance to respond “Accept” on a pair of participant and task. Given an individual and a task, every model *m* internally computes this value on the basis of a function $P _m : T \times I \rightarrow \mathbb {R}$, where *T* is the set of tasks and *I* is the set of participants. The expected prediction is then $\textit{max}(0,\textit{min}(P _m(t,i),1)$ given $t\in T$ and $i\in I$, ensuring a mapping to the domain [0, 1]. In the experiments, each task was presented to every participant exactly once; thus exactly one response is recorded per pair of person and task.

Before querying prediction function of a model, instantiated on the data of a participant, executes a **Pre-Training** function once. This function may optimize model parameters or data structures given data for the current individual and thus optimizes per participant. In the given setting, both Pre-Training and evaluation use data consisting of the complete set of task and participant pairs from the experiments with, including the participant’s response as well as task- and participant-level features.

In the following sections, we will present the algorithms implemented and tested to model a particular individual’s decision to reject or accept a news item. Section 4 will briefly introduce Dual-System-Theory and classical models that are in line with its statement that there is one “fast” and one “slow” pathway of cognition with different respective properties. Section 5 will show Heuristic models that assume a selection of simplifications underlying and shaping our cognition. Section 6 presents a selection of three well-performing approaches that do not strictly fall into any of these categories. These broaden up our presentation of cognitive theories, but are also more challenging to interpret.

## The Dual-System-Theory and Classical Models

A Dual-System-Theory (Kahneman, 2011) essentially describes that cognition is divided into two separate classes of processes, two “systems”: *System 1* activity is typically unconscious and describes intuitive processes and decision-making. Kahneman (2003) characterizes System 1 operations as “fast, automatic, effortless, associative, implicit [,] often emotionally charged; [...] governed by habit”. *System 2* accommodates intentional reasoning, such as decision-making through symbolic or logical inference. They are time-consuming, “serial, effortful” and assumed to be ”deliberately controlled” by the individual reasoner (Kahneman, 2003).

### Classical Reasoning

In the context of accuracy judgments about news items, the term classical reasoning as used by Pennycook and Rand (2019) and formulated by Kohlberg (1969) refers to the assumption that the extent to which people tend to think analytically, increases their likelihood to correctly classify “fake news” as misinformation and real news as real. In terms of the dual-process theory or Two-Systems View (Kahneman, 2003), measures of high system 2 activity such as the Cognitive Reflection Test should be correlated with correct classification of news items.

#### Implementation

1 $$P_{CR}(t,i)=\left\{\begin{array}{cc}\kappa_R+\alpha_R\ast CRT_i&t\;is\;real,\\\kappa_F+\alpha_F\ast CRT_i&t\;is\;fake.\end{array}\right.$$

*t* refers to a task, $CRT_i$ is the score achieved by participant *i* in the cognitive reflection test, addends and scaling factors $\kappa _R$, $\alpha _R$, $\kappa _F$, $\alpha _F$ are parameters determined in Pre-Training: The equations model a linear approximation of CRT and mean participant response for real and “fake news” items, respectively (globally over the set of all participants’ data). There are no free parameters. Yet notably, this model includes information on the truthfulness of the news item. Due to the correlation of truthfulness and participant responses (most items are categorized correctly) this gives it some advantage with respect to some other models presented below.

### Classical Reasoning & Reaction Time

Following the Dual-System Theory account, slower responses can indicate a usage of System 2 processes which are expected to give more consciously reflected and thus accurate classifications (Kahneman, 2011).

#### Implementation

This theory was implemented as an extension of the Classical Reasoning model: The reaction time *reac* of a person *i* on a given stimulus in task *t* is multiplied by a free parameter factor $\alpha$ and added to the expected response that the Classical Reasoning model yields.

$$\begin{aligned} P _{CR \& time}(t,i) = P _{CR}(t,i) * {\left\{ \begin{array}{ll} \alpha _R &{} t \ { is \ ''real'',}\\ \alpha _F &{} t \ { is \ ''fake''.} \end{array}\right. } \end{aligned}$$

$\alpha$ is optimized per individual.

### Motivated Reasoning

Motivated reasoning is related to the confirmation bias (Dawson et al., 2002). It proposes that individuals that are “motivated to arrive at a particular conclusion [...] construct a justification for their desired conclusion” actively (Kunda, 1990). Thus, under the assumption that motivated reasoning is a System 2 activity (Motivated System 2 Reasoning, MS2R) someone who thinks analytically would tend to classify information as correct that is favorable with respect to their own opinion and tend to reject information contradicting their previous convictions. In the given setting, higher System 2 activity would thus increase likeliness to accept news items the more they seem favorable for the political party the participant supports (Pennycook and Rand, 2019).

#### Implementation

A formalization of motivated reasoning must differentiate between 3 situations: The participant’s partisanship corresponds with the partisanship of the news item headline, the participant’s partisanship contradicts the news item partisanship or either one of these is unknown or neutral. In the latter case, the theory MS2R does not have a predictive implication; in the first two cases, the prediction respectively depends on the prevalence of analytical thinking in a participant. As the first case by MS2R is expected to yield a positive or at least stronger correlation with responding “Accept” than the second, linear parameters were introduced for both cases.

$$\begin{aligned} P _{MS2R}(t,i) = {\left\{ \begin{array}{ll} \kappa _C + \alpha _C * CRT_i &{} part_i = part_t, \\ \kappa _N + \alpha _N * CRT_i &{} part_i \not = part_t, \\ 0.5 &{} part_i \ { or } part_t { unclear}. \end{array}\right. } \end{aligned}$$

*C*, *N* stands for matching or non-matching partisanship of test participant and presented news item: $C =$ confirming view, $N =$ contradicting view of news item with respect to the persons political orientation. $part_t$ and $part_i$ scores (partisanship of news item and participant) are determined in experimental pretesting, which divided news items in neutral, favorable for Democrats and favorable for Republicans and questioned participants about their political orientation. $CRT_i$ is the cognitive reflection test score achieved by individual *i*. Addends $\kappa$ and CRT scaling factors $\alpha$ are free parameters optimized globally, for a fair comparison with *CR* in the above implementation, as well as the one in the study of Pennycook and Rand (2019).

### Suppression by Mood

Some experimental findings suggest that an intensive mood (be it positive or negative) affects people’s performance on tasks that require cognitive resources such as working memory (Oaksford et al., 1996). If we assume that classifying news items is a reasoning task that depends on working memory or related resources, the PANAS score appears interesting to implement a model for this hypothesis.

#### Implementation

As Suppression by Mood is not an own independent model but a hypothesis about reasoning processes, it was implemented as an extension of the Classical Reasoning model that depends on the imbalance of positive and negative affects in an individual:

$$\begin{aligned} P _{WMSUPR}(t,i) = P _{CR}(t,i) + \mid PANAS_p- {PANAS}_n \mid * {\left\{ \begin{array}{ll} \alpha _R &{} t \ { is \ real,}\\ \alpha _F &{} t \ { is \ fake.} \end{array}\right. } \end{aligned}$$

${PANAS}_p$ is the score on positive PANAS items, ${PANAS}_n$ the one on negative. $\alpha$ is a free parameter (optimized per individual) and $P _{CR}$ is the expected probability of the Classical Reasoning model as specified above.

### Improvement by Mood

Other studies actually show evidence that a somewhat negative mood is connected with more effortful and systematic processing (Forgas, 2017). Here, classification performance is expected to be influenced by an lower $\textit{PANAS}_p$ score and a higher one for ${PANAS}_n$.

#### Implementation

Like the suppression, Improvement by Mood was implemented as an extension of the Classical Reasoning model:

$$\begin{aligned} P _{WMIMPR}(t,i) = P _{CR}(t,i) + ({PANAS}_p-{PANAS}_n) * {\left\{ \begin{array}{ll} \alpha _R &{} t \ { is \ real,}\\ \alpha _F &{} t \ { is \ fake.} \end{array}\right. } \end{aligned}$$

${PANAS}_p$ and ${PANAS}_n$ are defined correspondingly as in model “Suppression by Mood”. $\alpha$ is a free, individually optimized parameter and $P _{CR}$ is the Classical Reasoning model’s expected probability.

## Heuristics for Reasoning

Reasoning with heuristics as prominently treated in the Adaptive Toolbox by Gigerenzer and Selten (2002), means using a set of simple, yet comparatively high performing (“satisficing”) rules. This bounded rationality approach was originally devised to facilitate high-risk decisions under time pressure; it acts both as a cognitive model and as an assistance tool for decision-making (Luan et al., 2011). Cognition with heuristics thus stems from a somewhat different branch of research than traditional cognitive models, i.e., the Two-System approach, as it is not always clear whether such heuristic indeed belong to System 1 or System 2 processes. However, individual heuristics also make claims about cognitive processing and thus can be formulated as modeling tools; also the models in this section do not require information on whether a news item is real or fake and thus can be considered candidates for actual cognitive processes — as opposed to, i.e., models based on the Classical Reasoning approach.

### Recognition Heuristic

This approach states that a stimulus is more likely be chosen, that is “accepted” by a person, if the person knows something about it, even if the information is not causally related to a reason to accept the stimulus. Here we assume that recognition values correspond to perceived familiarity measures of a news item from the pretest; this too was suggested in a study by Schwikert and Curran (2014).

#### Implementation

A formalization of recognition should yield “Accept” values for more familiar stimuli and “Reject” values for others; both a threshold model and a linear combination model seem plausible. Variants 1 (threshold) and 2 (linear):

$$\begin{aligned} P _{RECOG}(t,i) = {\left\{ \begin{array}{ll}1, &{}FAM_t > \kappa _i,\\ 0, &{}{else}. \end{array}\right. } \end{aligned}$$

$$\begin{aligned} P _{RECOG}^{{linear}}(t,i) = \kappa _i + \alpha _i * FAM_t \end{aligned}$$

$FAM_t$ is the perceived familiarity of stimulus *t* measured in a pretest, $\kappa _i$ and $\alpha _i$ are free parameters, optimized per individual.

### Fast-and-Frugal Decision Trees

**Fig. 2:**
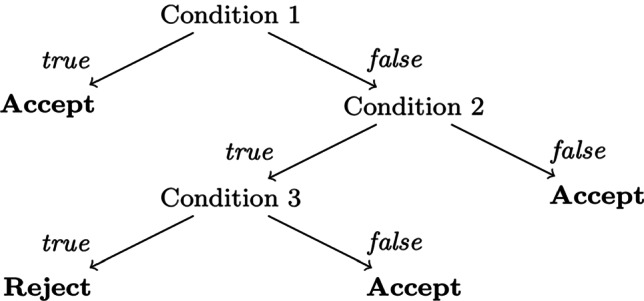
Example of a (binary) Fast-and-Frugal Decision Tree with three conditions

Fast-and-Frugal decision trees (FFTs) are intentionally simple, binary decision trees, where each node is connected to an output (Martignon et al., 2003). They are used in various disciplines for categorizing an object in order to make decisions with relatively little information, making them easy to construct and execute and thereby a class of heuristics (Martignon et al., 2008; Raab and Gigerenzer, 2015). Figure 2 shows an example FFT for a decision problem involving conditions on 3 features.

There are multiple strategies for selecting the ordering of features used for conditions and the respective direction of exits. Some strategies in the literature like ifan or dfan (Phillips et al., 2017) aim for optimal accuracy and they have been originally designed to facilitate decision-making in situations of limited time. Others such as Max or ZigZag (Martignon et al., 2003; Martignon et al., 2008) are derived from the Take-the-Best heuristic and optimize for best predictive performance; they do not consider conditional probabilities when selecting cues but only a greedy estimating measure of information gain.

#### Implementation

FFT generation algorithms were implemented following the specifications by Martignon et al. (2008) for Max and Woike et al. (2017) for ZigZag (Z+ variant; first exit node returns positive “Accept” result). They do not involve a depth limit. The features used per task and participant pair are all those listed as “relevant measured features” in the experiment description; contrary to all other models presented here, from the pretest both features measured for a current individual’s group (e.g., if an individual is identified as Republican, familiarity of a news item as aggregated value over all Republican participants) *as well as* the mean for both groups were used. The other models only take into account the first, more individualized value.


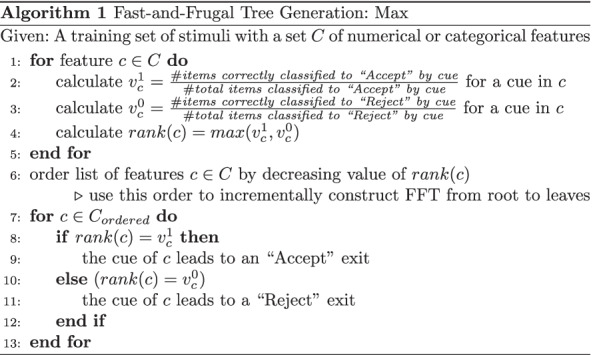


The ordering process in the for loop of Max (Algorithm 1) is considered an application of the Take-The-Best heuristic (Martignon et al., 2008). But Algorithm Max may yield “rake”-structured trees (trees where the ratio of “Accept” and “Reject” exits is strongly imbalanced) that might be unlike to cognitive representations. To avoid these, ZigZag enforces a binary alternating order of “Accept” and “Reject” exits. ZigZag uses the same Take-The-Best measure to determine feature/cue ranks, but the ordering of features is only the secondary specification for the resulting zigzag shaped FFT. Both FFT versions were optimized globally to avoid overfitting per person and retain interpretability: Rather than a multitude of trees (one per participant), a single one was trained for each whole dataset.

As an example, the first four queues for each model are shown in Algorithms 2 and 3 optimized on the dataset from Experiment 3.
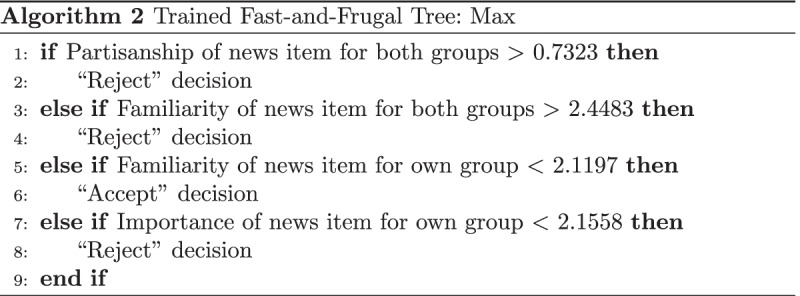

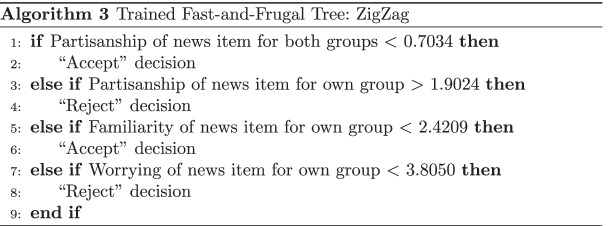


## Other Models

### Weighted Sentiments

We included a linear combination of sentiments that were globally optimized as an additional model. The sentiment analysis was conducted using the Empath library for Python (Fast et al., 2016), which assigns words in a text to pre-built categories. These are generated by deep learning methods over a large volume of text from modern fiction. To avoid overfitting to headlines and again to keep track which sentiments are relevant for classification, just a selection of the most frequently occurring sentiment categories was used here.

The idea behind this model is that people — maybe consciously, but rather unconsciously — are affected by the mood an article headline purports, when deciding whether to believe it. Also depending on what topics a sentence touches upon, it may either seem believable or unlikely for a particular reader with individual preferences.

#### Implementation

$$\begin{aligned} P _{SENTIMENTS}(t,i) = {\left\{ \begin{array}{ll}1, &{}\sum _{c=1}^{n} s_{c}(t) * \alpha _{c,i}~ \ge 0,\\ 0, &{}\sum _{c=1}^{n} s_{c}(t) * \alpha _{c,i}~ < 0. \end{array}\right. } \end{aligned}$$

$\alpha _{c,i}$ are free weighting parameters (optimized per individual) for a set of *n* sentiment measures $s_{1}(t) \dots s_{n}(t)$ per headline of task *t* and for participant *i*. 10 sentiments features were weighted for each headline. So, this model is a linear combination of the sentiments

The headlines in each experiment yield different numbers of sentiment categories with non-zero values for at least one headline. Allowing all these measures to be part of the model’s calculation would make the model too complex and make it overfit easily; sentiment combinations could map too precisely onto news items rather than allow the model to generalize on news item groups that have sentiment values in common. However, choosing a number significantly less then 10 would render the remaining sentiments ineffective for discrimination of items. The constant number 10 was chosen as a reference value just to ensure comparability among experiments and keep interpretability. For each of the two datasets, the 10 sentiments most frequently detected in respective news item headlines were selected. These were, for Experiment 1 and 2: *dispute, fight, war, strength, politics, aggression, government, communication, traveling, leader* and for Experiment 3: *power, wealthy, banking, valuable, giving, law, payment, economics, money, government*.

### By Partisanship

All of the theories presented above do not take into account the actual partisanship of a participant, but at best use this information to classify them as rather Republican or rather Democrat (e.g., in the S2MR model). This current model takes reference to the thesis that membership of one of these groups is an indication for higher susceptibility to misinformation; the claim that people sympathizing with the Republican party are worse at classifying news items.

#### Implementation

$$\begin{aligned} P_{PARTY}(t,i) = {\left\{ \begin{array}{ll} \kappa _R+ \alpha _R*{part}^{\prime }_t &{} t \ {is \ ''real''}, \\ \kappa _{F}+ \alpha _{F}*{part}^{\prime }_t &{} t \ {is \ ''fake''}. \end{array}\right. } \end{aligned}$$

${part}^{\prime }_t$ refers to the distinctive partisanship of task *t*, that is small values for a news item classified as Democrat-partisan in the pretest and high values for an item classified as Republican-partisan; $\alpha _R, \alpha _F$ and $\kappa _R, \kappa _F$ values are free parameters fitted globally.

However, on optimization, it turned out that this model actually converged with a model that always yields correct classifications for a news item; “real” news are always accepted and “fake” items always rejected. Thus, this model’s claim could not be verified and it was omitted from further evaluations.

### Van Bavel

This model is included as an up to date alternative to particularly the motivated reasoning model presented above: In fact, modern research on motivated reasoning does not necessarily support the theories that the current implementation of motivated reasoning is based upon, such as in Kahan (2012); Kahan et al. (2017); e.g., the studies Van Bavel and Pereira (2018); Pretus et al. (2021) put forth criticism of this account. Two studies reanalyzing the original work by Pennycook and Rand (2019) argue that the influence of partisanship was in fact underestimated there (Rathje et al., 2020; Gawronski, 2021). Thus, to assess an alternative identity-based approach to motivated reasoning, we base this model on Van Bavel’s account of misinformation processing under the influence of participants’ identity (Van Bavel et al., 2020), which lists main risk factors of falling for fake news. Among these, *intellectual style*, *memory (familiarity)*, *partisan bias* and *polarization* can be mapped to features that were measured in the given experiments.

#### Implementation

$$\begin{array}{ll}P _{{vanBavel}}(t,i) =\\\alpha *{FAM}_{t} + {\left\{ \begin{array}{ll} \kappa _R +\beta _R*{part}_i + \gamma _R*{part}^{\prime }_t &{} t \ { is \ ''real''} \\ \kappa _F +\beta _F*{part}_i + \gamma _F*{part}^{\prime }_t &{} t \ { is \ ''fake''} \end{array}\right. } + {\left\{ \begin{array}{ll} \kappa _p &{} part_i = part_t, \\ 0 &{} {else}. \end{array}\right. }\end{array}$$

$\alpha , \beta _R, \beta _F, \gamma _R, \gamma _F$ are free weighting parameters and $\kappa _R, \kappa _F, \kappa _p$ offset parameters, all optimized per individual. $CRT_i$ stands for individual *t*’s CRT score and ${FAM}_t$ is news item’s *t* familiarity (aggregated among members of individual *i*’s group). ${part}_i$ is the absolute partisanship as in the models above; the degree of a news item’s partisanship, regardless of the party endorsed. ${part}'_t$ refers to the distinctive partisanship of task *t*, that is small values for a news item classified as Democrat-partisan in the pretest and high values for an item classified as Republican-partisan. The expression ${part}_i = {part}_t$ means that task *t*’s partisanship corresponds to that of person *i*.

Noticeably, just as Van Bavel takes reference to numerous factors in his model of misinformation acceptance, the features it depends upon are numerous. This makes this model more challenging to interpret than some of the heuristic models above, even given high prediction accuracy.

## Predictive Performance of Models

Our evaluation approach focuses on testing, how well models predict a response of each individual participant. This allows to possibly falsify models and compare their performance.

### CCOBRA

To ensure a modeling evaluation standard, we used the CCOBRA framework^6^ that has been recently proposed (Ragni et al., 2019) and also used for modeling syllogistic reasoning, spacial reasoning and conditional reasoning tasks (e.g., (Brand et al., n.d.), (Friemann and Ragni, 2018), (Todorovikj and Ragni, 2021) respectively). It is benchmarking tool designed for evaluating implementations of cognitive reasoning models on their predictive quality. Given experimental data from a study of decision made by test persons, CCOBRA provides for models an experiment like test scenario and compares the predictions of cognitive models with decisions made by individuals. In particular, information about a specific test person can be taken account in the models’ predictions.

In our case, CCOBRA provides news item headlines together with information about a given test person and expects a response from the model. Then for each pair of participant and task, the framework compares the participant’s actual reply with the prediction given by the model. Cognitive models in the framework can make use of three types of functions: Pre-Training, Prediction and Adaptation. *Pre-Training:* Each cognitive model can use data from the training set. The training dataset can either contain data from the current individual (“optimization per participant”) or all participants (“global optimization”). *Prediction:* Given a news item headline, an initialized and trained model is queried to return a prediction. With respect to the current research question this means: Will the given individual accept a given news item as truthful or will they reject it as inaccurate? The Prediction function then by default returns true or false respectively. Every call of the Prediction function is by default followed by a call of the Adaptation function, as typically the presentation of a new stimulus (news items) may result in a change of internal parameters of a model.

For this study of models that do not have the capacity to store provided correct responses, a setting was used where test and training datasets for each modeled individual are same. As the experimentally known decision data for each individual is very limited (10, 12 and 18 fake and real distinct items in Experiments 1, 2 and 3 respectively), this is a more reasonable approach to train the model parameters than to split these already small datasets into distinct training and test sets with an even smaller number of items — very small, but differently sized training or test sets would also raise further issues in terms of statistical comparability of the experiments. Due to the simplicity of these models, the decisions of each individual can still not be modeled perfectly. The results obtained by this method indicate, which of them are to what extent capable of accounting for a person’s decisions, even given the full known dataset.

Thus, to really put the CCOBRA framework to use as it is supposed to and yields most informative results, non-aggregated data, that is experimental records for each individual and task outcome are required. Then, either models can be tested that use all known information about task and person, or models that do not require knowledge of the *true* response to a task (not always an applicable description, but in this case the true categorization of news items as fake or real). Models belonging to both of these groups were part of this study. Yet, while each can provide valuable findings, the latter group may be regarded more interesting, as it more closely models the actual processes of a reasoner in the experiment; they too do not know, whether a news item is indeed fabricated or authentic and can not rely on this information in their reasoning. A model that does this would therefore not strictly be counted as a model of cognitive processes; however, as these too are relevant and descriptive of the observed processes, they were included in this study (e.g., the Classical Reasoning model).

Further, it is quite easy to imagine further extensions for given models, such as a CR model that also takes into account familiarity or partisanship. However, as such potential combinations would be practically unlimited, we decided to stick to models that implement one consistent theory and can be reasonably interpreted in their workings; the more parameters and features are included in a model’s prediction, the more difficult it gets to infer their respective significance and the overall character of the model’s way of calculating predictions. Yet, an approach to study combined or “hybrid” models is shown in Sect. 7.4.

### Parameter Fitting

Though CCOBRA defines an architecture that leaves the choice of global pre-training (parameter fitting) of a model, or separate pre-training for all test persons respectively and subsequent individual modeling, it leaves the concrete fitting algorithm to be specified by each model. In the present study, to ensure comparability among the models implemented, the same method of fitting free parameters was used to train all presented models: A basin-hopping algorithm that performs local optimization at 200 random perturbations around given initial coordinates; these were set to 0. A hyperparameter that describes that randomness of such perturbations, the temperature hyperparameter *T* was set to 5 (standard is 1). The exact working of the used implementation is described in (Wales and Doye, 1997); it is part of the Python module scipy.optimize.

Some modules that do not contain free parameters or because of their characteristics can not be trained in the way described, e.g., FFT models, were fitted with different procedures, as described in the respective Implementation sections.

### Evaluation

**Fig. 3:**
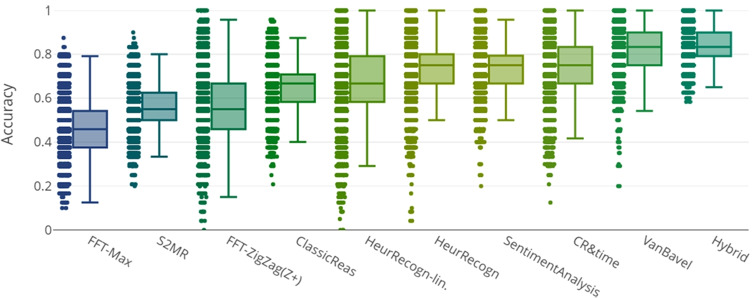
The predictive accuracy of each model for each individual participant (represented as a dot) in Exp. 1 and Exp. 2

**Fig. 4:**
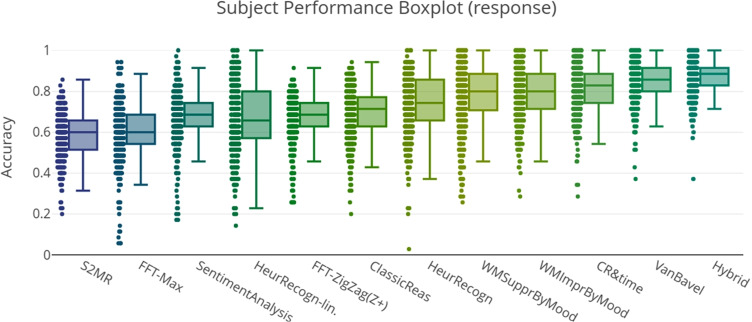
The predictive accuracy of each model for each individual in Exp. 3

We assessed the predictive accuracy of the models given the respective experimental data for both settings: The graphical visualizations in this section provide as first overview (Figs. 3 and 4) — tables with exact values follow below.

The models of Suppression and Improvement by Mood were only included in evaluations of Experiment 3, as data for Experiments 1 and 2 does not include PANAS score measures.

**Table 1:** Predictive accuracy of models in Exp. 1 and 2

Model	Predictive Performance
Hybrid Model (best)	0.84, $MAD=0.05$
Van Bavel Model	0.83, $MAD=0.07$
Sentiments	0.75, $MAD=0.08$
Recognition Heuristic	0.75, $MAD=0.08$
CR&ReactionTime	0.75, $MAD=0.08$
Recognition Heuristic-Lin.	0.67, $MAD=0.08$
Classical Reasoning	0.65, $MAD=0.05$
FFT Zigzag (Z+)	0.62, $MAD=0.12$
S2 Motivated Reasoning	0.55, $MAD=0.03$
FFT Max	0.46, $MAD=0.08$
Data Baselines	
Correct Categorization	0.71, $MAD=0.08$
Always “Reject”	0.6, $MAD=0.10$
Random	0.5, $MAD=0.00$

Table 1 shows predictive performance and other measures of implemented models for the datasets of Experiments 1 and 2, Table 2 for Experiment 3. FFT models were not optimized per participant, as for the very limited number of tasks in these experiments, constructing a decision tree per participant appears to be very prone to overfitting. Their Predictive Performance measures refer to a globally optimized version that nevertheless performed comparatively well.

**Table 2:** Predictive accuracy of models in Exp. 3

Model	Predictive Performance
Hybrid Model (best)	0.87, $MAD= 0.04$
Van Bavel Model	0.86, $MAD=0.06$
CR&ReactionTime	0.81, $MAD= 0.06$
WM-Improvement by Mood	0.77, $MAD= 0.09$
WM-Suppression by Mood	0.77, $MAD= 0.09$
Recognition Heuristic	0.74, $MAD= 0.09$
Classical Reasoning	0.71, $MAD= 0.06$
FFT Zigzag (Z+)	0.69, $MAD= 0.06$
Recognition Heuristic-Lin	0.66, $MAD= 0.11$
Sentiments	0.69, $MAD= 0.06$
FFT Max	0.60, $MAD= 0.06$
S2 Motivated Reasoning	0.59, $MAD= 0.06$
Data Baselines	
Correct Categorization	0.79, $MAD= 0.09$
Always “Reject”	0.57, $MAD= 0.09$
Random	0.50, $MAD= 0.00$

As seen in Tables 1 and 2, most models indeed perform distinctively better than random. While Fast-and-Frugal trees comparatively yield reasonable but not highest predictions, the ZigZag (Z+) version interestingly, much outperforms Max for the present data. This was also the case in the study by Martignon et al. (2008). Further investigation showed that Max in fact ended up replying “Reject” to most of the items presented.

The sentiment analysis model preformed distinctively better on the dataset of Experiments 1 and 2 than on Experiment 3. This could be explained by a less specific selection of sentiment keywords in Experiment 3 (18 fake and 18 real as compared to respectively 10 or 12 fake and real in Experiments 1 and 2). As the same number of sentiments was weighted by the model in both settings, the larger amount of different headlines in total probably resulted in a more ambiguous set of sentiments as can be seen in the listing in the respective model description in Section 6.1: In Experiment 3, the words generally describe concepts from the sphere of public policy and finance, whereas Experiment 1 and 2 yielded sentiments that are more specific; often also more emotionally laden.

Recommender models (a type of predictors that do not model cognitive processes but has access to the decision data of all other experiment participants and bases predictions on decisions of similar individuals) proved difficult to optimize, as they tended converge to a model that gathers prediction means over all participants for every item: Instead of selecting a subset of features to specify a specific “similarness” of some individuals with others, it performed best, the more people were pooled as “similar” and the prediction made as the mean value of their responses. As in 72% (Exp. 1, 2) resp. 79% (Exp. 3) of tasks participants categorized news items correctly, such recommenders applied this probability to each news item and as a result always yielded “Accept” for real news an “Reject” for fake news — as does the baseline model Correct Categorization.

### Hybrid Models

A hybrid model combines individual models by detecting for each participant *p* the reasoning model *m* that performs best and using *m* for predicting (respectively fitting) all data points of tasks presented to *p*. Then an overall predictive performance of the decision of an individual participant of **0.84** (Exp. 1, 2) resp. **0.87** (Exp. 3) with a notable effect on the $MAD = 0.05$ resp. 0.04 can be reached by an ensemble model approach. Note that the difference between these upper bounds in both settings is relatively small.

The Van Bavel-inspired model and CR&time model ranked first, resp. second in both settings. This is highly interesting considering their different architecture; the only feature they both use is CRT. It is too interesting, that the hybrid model just closely outperformed them in both settings. This suggests that the Van Bavel model in fact almost always outperforms any other model in this selection. Though an impressive finding, this leaves many questions unanswered due to the properties of this theory that were hinted to above (and that CR&time also shares): We have now shown on the given experimental data that the features this model uses, that is, the risk factor Van Bavel’s theory states do indeed play a significant role on “Accept” decisions. However, the model strongly relies on the information, whether a news item is in fact fake or not — while we can describe what decision will be made, we can still only speculate, which processes actually happen in the test participants from the *cognition* point of view.

Thus, to see if the other, i.e., cognitively interpretable models can offer an alternative explanation to the Van Bavel model, we run the hybrid model again over all models but that, which yielded median results 0.80 (${MAD}=0.05$) for Exp. 1 and 2; and 0.86 (${MAD}=0.06$) for Exp. 3. These are only slightly lower than for the original hybrid, while still significantly higher than any other individual mode.

This discrepancy between individual models and their ensemble now alternatively points to the possibility that different “cognitive tools” are employed by different participants. For an indication, which models could best fit such a “tool” approach, the same evaluation was further conducted for hybrid models that were allowed to choose from a set of only two models — again excluding the Van Bavel model to focus on potential cognitive processes at this time. That is, for every combination of any two regular models, each participant was evaluated with the one model that yielded the more accurate prediction of the two selected (sub-)models. Tables 3 and 4 show the five best predictive performances of such two-model hybrids and their composition over outputs of all models optimized per participant. Note the high prevalence of recognition-based models among the best-performing pairs. These are mostly paired with CR (CRT-based) models or, i.e., in Table 3, also the Sentiment model that was among the most accurate predictors in the dataset of Exp. 1 and 2.

**Table 3:** 2-Model Hybrid Combinations: Experiments 1 and 2

Hybrid Model	Mean	$\sigma$	Median	MAD
Sentiment & CR&Rt	0.79	0.09	0.79	0.06
Recognition & CR&Rt	0.79	0.10	0.79	0.06
Recognition & Sentiment	0.77	0.10	0.79	0.07
Recognition-lin. & CR&Rt	0.77	0.10	0.79	0.08
Recognition-lin. & Sentiment	0.76	0.09	0.75	0.06

**Table 4:** 2-Model Hybrid Combinations: Experiment 3

Hybrid Model	Mean	$\sigma$	Median	MAD
Recognition & CR&Rt	0.83	0.09	0.83	0.06
Recognition & WM-Impr	0.82	0.10	0.83	0.06
Recognition & WM-Suppr	0.82	0.10	0.83	0.06
CR&Rt & Sentiment	0.82	0.09	0.83	0.06
CR&Rt & Recognition-lin.	0.82	0.10	0.83	0.06

### Correlated Features and Models

Apart from comparing the prediction accuracy of models, it is still necessary to clarify the relations models have among each other. Do models behave very similar in their predictions or are there selections of models that yield different results but are still quiet accurate? The first case would point to a single cognitive pathway that is just modeled by different formulations, whereas the second case would suggest that in fact different types of processes are used by the experiment participants in their decision-making.

Thus, this section gives an overview on how models and selected features of a news item-test particular pairing are correlated: The first is to assess the distinctness of model workings, the latter is to visualize the potential impact of singled out features on a model’s prediction.

**Fig. 5:**
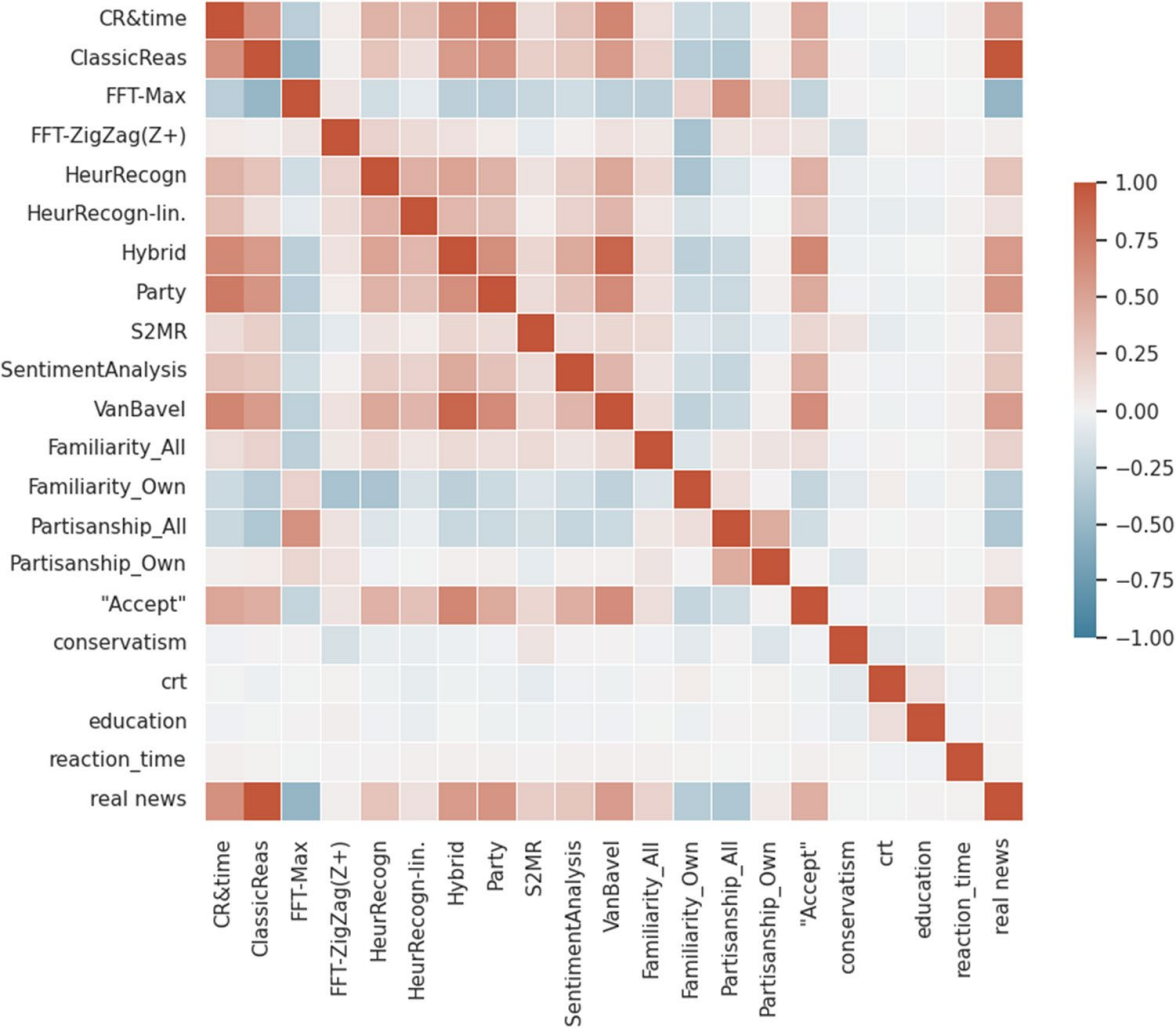
Correlation of model predictions and task, participant features in Exp. 1 and 2

**Fig. 6:**
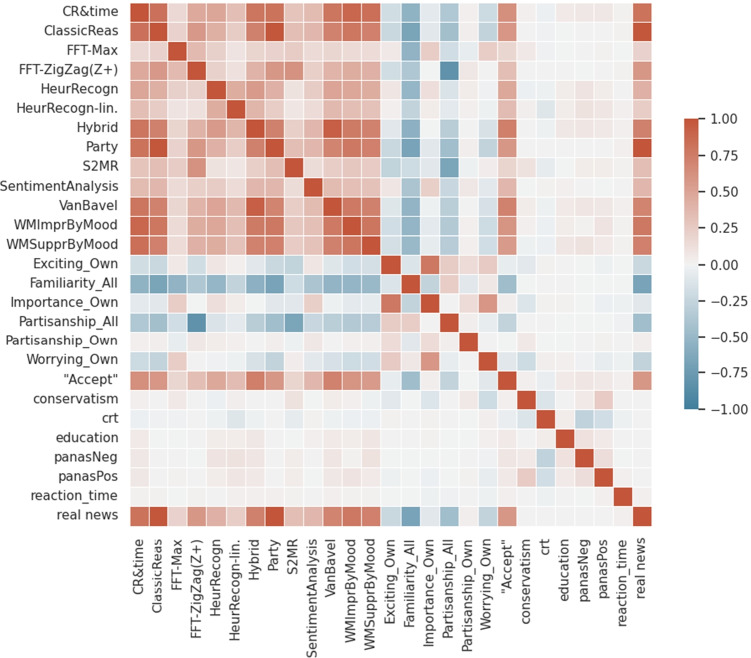
Correlation of model predictions and task, participant features in Exp. 3

Figures 5 and 6 show correlations of features related to the task and participant features and model predictions; features marked with “_All” refer to the assessment of a news item by both persons identified as Republican and Democrat — this information stems from the pretests. In the tables, “_Own” refers to values measured for the participant’s own group (Conservative or Republican) for a news item. “real news” indicates where the presented news item was authentic (real) and “Accept” refers to the actual classification decision of the experiment participant.

Firstly, not all individual models are correlated strongly among each other, showing that for modeling reasoning with news items, different strategies seem to emerge successful.

Though familiarity already correlates with participant responses (“Accept”), the correlation of the recognition models, especially the threshold model, is higher, thus pointing to the legitimacy of a threshold formula. Correlations of features conservatism, CRT, education, reaction time and PANAS with responses and most models are rather role, although part of some well-performing models. Besides, the strong correlations between “Exciting” and “Importance” values as well as “Importance” and “Worrying” are notable. Further, the strong correlation of partisanship with FFT Max and decorrelation with FFT ZigZag predictions is interesting and might model different reasoning, however with FFT ZigZag the significantly better-performing model.

The strong correlation of Suppression by Mood with CR & Reaction Time predictions could indicate that the processes modeled are related or that the models might rely on different manifestations of the same process: Working Memory access might be more or less efficient depending on both mood and invested reaction time.

Finally, it is interesting to observe that the Hybrid model is in both experimental settings best correlated with the Van Bavel model and CR & ReactionTime. After that it correlated higher with CR and the threshold implementation of the recognition heuristic than with the linear implementation. While in Exp. 1 and 2, it also strongly correlates with the SentimentAnalysis model, in Exp. 3 it relies less on it and only slightly more than on the Mood models included here.

## Discussion and Conclusion

The results of this paper allow us to make a few assumptions about modeling misinformation processing. First, the evaluations of models CR and S2MR reflect a result achieved by Pennycook and Rand (2019); CR turns out to yield much better predictions than the motivated reasoning account. Although as expected CR outperformed motivated reasoning, yet the implemented S2MR model does have a prediction accuracy higher than random (see Tables 1, 2). The presented individualized modeling approach with CCOBRA thus confirms that CR accounts reasonably well for people’s reject or accept decision of news items, but indicates that the motivated reasoning account is not completely unrelated to the decision-making behavior observed.

On another note, it should be mentioned that the current experimental results are likely to be limited to a political situation as in the United States, where the experiment participants were based. Other studies indicate, that in other place, identity and partisanship divisions are less clear cut, e.g., as explicated by Faragó et al. (2019) with respect to eastern Europe.

Further we have shown high predictive accuracy for additional models, some of which are extensions of the CR model taking into account more features known from the experimental data. An interesting finding is that a participant’s perceived familiarity with a news item appears to play a major role in judging its accuracy. The recognition heuristic relies on this measure and achieves successful predictions. This is consistent with the finding that repeated exposure to a news item increases its perceived accuracy (Pennycook et al., 2018).

Further, sentiment analysis provided interesting findings: While yielding very good results, it seems to suggest that word fields implying negative rather than positive emotions in some way receive more “Accept” responses

Finally, the recommender optimized in a way that includes many participants rather than just a few specific ones and gets close to a model that always classifies news items correctly. This may indicate that a linear combination of the measured features does not reliably explain participants’ “Reject” vs. “Accept” classification behavior and their classification success; instead, pooling decisions of many different individuals leads to both comparatively successful prediction and often accurate classification of a news item. In conclusion, numerous heuristic models perform reasonably well in explaining news item acceptance decisions of a participant and improving predictions of classical reasoning theory, even without information on whether the item is misinformation or not. Here, the features most significant for a participant’s acceptance decision appear to be perceived familiarity, partisanship, importance, perhaps “thrillingness” of a news item, and to a lesser extent, time spent on the decision.

In this study we did not evaluate, whether any models are pareto-optimal in comparison to others — that is, whether they predict better for all participants. This could be a question for further investigation; however, methods would need to be devised that can perform this check while accounting for inevitable outliers.

The model informed by Van Bavel’s theory of misinformation processing offers a good descriptive accuracy, but leaves a number of questions open as to cognitive processes. Many other models perform well although uncorrelated, which indicates that there may be different kinds of underlying processes in the present kind of decision-making in a single individual or among groups that have not been identified yet. The improvements in predictive performance achieved with some hybrid models support this interpretation of an adaptive toolbox of strategies to evaluate news on the individual level — of course, it is fairly expectable that a model that can choose among a selection of other models outperforms them, but the observation that already two-model combinations reach almost the same predictive accuracy is notable. Indeed, on the one hand it seems plausible that a reasoner has a limited number of pathways they choose from in cognitive processing. On the other hand, some of the shown 2-model hybrids are consistent with the paradigm of Dual-System-Theory.

Also a participant’s mood seems to be an important predictor; yet, in our setting both models perform almost equally good, pointing to either the presence of different ways mood can influence news acceptance, or that an optimal way to integrate the PANAS score in a model of this kind remains yet to be found that will outperform these models.

Our findings also offer avenues for successful interventions to improve the accuracy of online decisions by considering the decision-making process and its context explicitly, going beyond third-party fact-checking. For example, if familiarity is an important consideration, providing related articles alongside news could be a way forward; encouraging deliberate decisions through friction or pooling judgments could also be promising avenues (Lorenz-Spreen et al., 2020). Further studies could focus on clustering participants by parameters fitted and implement more sophisticated strategies for choosing among sub-models in hybrid models than max-pooling, e.g., decision trees for selecting models by news item or experiment participant features, which could give indices, when a particular decision strategy is performed. Another idea could be to include different computational measures concerning the content of a news item, such as image recognition or semantic natural language parsing that were beyond the scope of this paper.

Evaluating the power of models in a predictive setting is a new, rigorous, and promising method to systematically test cognitive models. At the same time, it provides a step forward to systematically construct new and better models to capture the specifics of the individual participant and automatically enriching an adaptive model toolbox.

## Data Availability

Data and material for experiments are publicly available: Experiment 1: https://osf.io/h2kms/. Experiment 2: https://osf.io/5dsf8/. Experiment 3: https://osf.io/f5dgh/. Pretest for Experiments 1 and 2: https://osf.io/p6u8k/. The pretest for experiment 3 was received directly from the author and is, to our knowledge, not yet fully published.

## Footnotes

1 Publicly accessible dataset: https://osf.io/h2kms/

2 Publicly accessible dataset: https://osf.io/5dsf8/

3 Publicly accessible dataset: https://osf.io/f5dgh/

4 Publicly accessible dataset: https://osf.io/3mnpb/

5 Dataset received directly from author, not yet fully published as of August 2021.

6 https://github.com/CognitiveComputationLab/ccobra

## Code Availability

Code for models and evaluation is fully available (https://github.com/borukhd/Individual-Accept-Misinformation).
